# Editorial: Gut dysbiosis-induced systemic inflammation in neurological diseases and disorders

**DOI:** 10.3389/fimmu.2024.1437651

**Published:** 2024-06-05

**Authors:** Pradeep Kumar Shukla, Shirin Fatma, Mohammad Moshahid Khan

**Affiliations:** ^1^ Department of Pathology, St. Jude Children’s Research Hospital, Memphis, TN, United States; ^2^ Department of Structure Biology, St. Jude Children’s Research Hospital, Memphis, TN, United States; ^3^ Department of Neurology, College of Medicine, University of Tennessee Health Science Center, Memphis, TN, United States

**Keywords:** microbiome, gut-brain axis, machine learning, obesity, IBD, alzheimer’s disease, multiple sclerosis, autism

The human gut microbiome consists of trillions of microorganisms, encompassing several species of bacteria, viruses, and fungi. It plays a significant role in both human health and disease pathogenesis. Acute and chronic shifts in the microbiome occur in response to multiple stimuli including environmental, emotional, and physical stress. The alteration of the gut microbial population and its impact on several neurological diseases including Alzheimer’s disease (AD) have been established in recent years (1–6). The gut-brain axis represents the synergistic pathway linking the gut microbiome and the brain, facilitating communication through neural, endocrine, and immune mechanisms (7–10). Therefore, understanding how the gut-brain axis influences disease conditions may provide novel insights into disease pathobiology and identify novel therapeutic targets.

Verhaar et al., investigated the connections between gut microbiota composition and AD biomarkers, employing machine learning models. Drawing from the diverse Amsterdam Dementia Cohort, the authors meticulously analyzed fecal samples from 170 patients at different stages of cognitive decline, ranging from AD to mild cognitive impairment and subjective cognitive decline. They delineated predictive relationships between gut microbiota composition and markers such as cerebrospinal fluid amyloid-beta and phosphorylated tau, in addition to visual MRI scores indicating structural changes in the brain. Major findings of the study were the associations with specific microbial species, such as *Clostridium leptum*, whose higher abundance correlated with increased odds of amyloid positivity, while others like *Lachnospiraceae* spp. and *Roseburia* hominis hinted at protective effects against AD pathology. These findings not only underscore the complexity of the gut-brain axis but also present novel avenues for intervention and treatment. This study is representative of the translational potential of interdisciplinary collaboration in unraveling the complexities of neurological disorders.

In a similar vein, the study by Shahi et al., shed light on the impact of obesity-induced gut dysbiosis on the severity of multiple sclerosis (MS). Through meticulous experimentation, they revealed the profound influence of gut microbiota composition on MS pathobiology, offering insights into potential therapeutic targets. The authors showed the mechanisms underlying obesity and the severity of MS using high-fat diet (HFD)-induced obese HLA-DR3 transgenic mice, a model that mimics key aspects of MS in humans. These researchers also showed a correlation between gut microbiota, obesity, and MS severity. The findings provide new insights into HFD-induced obesity, gut microbiota composition, and MS pathobiology. Notably, obese mice exhibited gut dysbiosis, characterized by alterations in microbial composition and metabolic pathways. The abundance of *Proteobacteria* and *Desulfovibrionaceae* bacteria increased, accompanied by the regulation of microbiome-based metabolic pathways, including those implicated in hydrogen sulfide biosynthesis. This study shows the way for personalized interventions aimed at mitigating MS severity.

Berg et al., explored the role of melanin in neurodegenerative diseases, providing a fresh perspective on disease pathogenesis. Their exploration of the multifaceted roles of melanin highlighted its significance beyond superficial functions and offered insights into its potential implications for AD, Parkinson’s disease (PD), and Lewy Body Dementia (LBD). Melanin, it appears, is far more than a passive bystander in the body’s landscape. This study presented a convincing narrative of melanin’s multiple roles, in the immune system, metal absorption, thermo regulation, and energy transduction. Notably, melanin’s presence is pervasive, spanning various tissues such as the brain, heart, arteries, and even within individual cells as precursors. The authors explored the loss of melanin and its implications for disease pathogenesis. Drawing parallels between diseases characterized by melanin depletion, such as vitiligo, and neurodegenerative conditions like AD, PD, and LBD, the study posits a unifying hypothesis. At its core is the notion that melanin, under normal healthy conditions, retains and channels energy absorbed from electromagnetic radiation to fuel cellular processes. However, the loss of melanin disrupts this delicate energy balance, leading to a cascade of cellular dysregulation.

The connection between the gut and the brain has long been studied but the interplay between gastrointestinal health and neurological disorders is complex, with mounting evidence suggesting bidirectional links between inflammatory bowel disease (IBD) and neurodegenerative conditions.

A meta-analysis by Zong et al., studied the association between IBD and several neurodegenerative disorders. The authors provided a comprehensive summary of the bi-directional prospective relationship between IBD and neurodegenerative disorders through a detailed analysis of longitudinal studies. Through a thorough bibliographic search spanning multiple databases, the researchers meticulously curated data from 27 studies to provide a comprehensive overview of the relationship between IBD and neurodegenerative disorders. This meta-analysis revealed a heightened risk of developing neurodegenerative disorders in individuals with IBD. Across the spectrum of neurodegenerative conditions, including AD, MS, and PD, IBD patients exhibited increased susceptibility. Additionally, the study uncovered intriguing insights into less explored connections, such as the elevated risk of amyotrophic lateral sclerosis and multiple system atrophy in IBD patients. Findings from this study show an association between the gut-brain axis, highlighting the potential role of gastrointestinal health in influencing neurological outcomes.

A review by Feng et al. demonstrated the potential synergies between repetitive transcranial magnetic stimulation (rTMS) and modulation of the gut microbiota as an effective treatment strategy for autism spectrum disorders (ASD). rTMS, a noninvasive neuromodulatory technique, has shown promise in various psychiatric disorders, but its efficacy in ASD, particularly in addressing gastrointestinal symptoms and leveraging the gut-brain axis, has been relatively unexplored. This review provided a comprehensive overview aimed at bridging the gap between rTMS therapy and gut microbiota modulation in the context of ASD. Through a rigorous analysis of the literature, the review highlighted the potential therapeutic benefits of integrating rTMS with gut microbiota interventions, offering a targeted approach tailored to the unique needs of individuals with ASD. Central to the review is the recognition of the gut-brain axis as a pivotal mediator in ASD pathogenesis. Dysregulation of the gut microbiota is linked to ASD, with profound implications for neurological functioning. By exploring the interplay between rTMS and gut microbiota modulation, Feng et al. uncovered a potential symbiotic relationship in which neuromodulation may exert therapeutic effects via the gut-brain axis. Moreover, the review underscores the importance of understanding the underlying mechanisms driving this synergy. By elucidating the intricate pathways through which rTMS and gut microbiota interventions overlap, researchers may uncover novel targets for ASD treatment, offering hope for improved outcomes and quality of life for individuals affected by this condition.

Taken together, these studies exemplify the power of interdisciplinary collaboration in unraveling the complexities of neurological disorders and the gut-brain axis. As we stand at the dawn of a new era in neurological research, these investigations are uncovering critical insights into disease pathobiology, offering hope for innovative therapies and a brighter future for individuals affected by these debilitating diseases.

## Author contributions

PS: Writing – original draft, Writing – review & editing. SF: Writing – original draft, Writing – review & editing. MK: Writing – original draft, Writing – review & editing.
